# Structure of the giant RNA polymerase ejected from coliphage N4

**DOI:** 10.21203/rs.3.rs-7746245/v1

**Published:** 2025-10-21

**Authors:** Nathan F. Bellis, Ravi K. Lokareddy, Mikhail Pavlenok, Stephanie L. (Cooper) Horton, James L. Kizziah, Francesca Forti, David A. Schneider, Michael Niederweis, Federica Briani, Gino Cingolani

**Affiliations:** 1 Department of Biochemistry and Molecular Genetics, University of Alabama at Birmingham, 1825 University Blvd, Birmingham, AL 35294, USA; 2 Department of Microbiology, University of Alabama at Birmingham, 845 19th Street South, Birmingham, AL 35294, USA; 3 Institutional Research Core Program (IRCP), University of Alabama at Birmingham, 845 19th Street South, Birmingham, AL 35294, USA; 4 Dipartimento di Bioscienze, Università degli Studi di Milano, Milan, Italy

## Abstract

*Schitoviruses* are widespread prokaryotic viruses that encapsidate a giant (~3,500-residue) virion-associated RNA polymerase (vRNAP). During infection, vRNAP is expelled into Gram-negative bacteria, along with two additional ejection proteins, to assemble a transient DNA-ejectosome that becomes transcriptionally active, initiating viral replication. Here, we present an integrative structural analysis of the coliphage N4 vRNAP (gp50). We find that this 383 kDa enzyme is a multi-domain, single-chain RNA polymerase, structurally distinct from both compact single-chain RNAPs and large multi-subunit holoenzymes. vRNAP is composed of loosely connected domains and exhibits an intramolecular mode of allosteric regulation through its C-terminal domain. Comparative analysis of intact and genome-released virions identified gp51, which forms an outer-membrane complex, and gp52, which assembles a periplasmic tunnel. These proteins generate heterogeneous pores that facilitate the release of vRNAP. We further uncover a signaling hub in the phage tail, composed of the receptor-binding protein, tail tube, and tail plug, that detects receptor engagement and orchestrates the release of ejection proteins. We propose that the beads-on-a-string architecture of vRNAP enables the translocation of megadalton-scale protein complexes through the ~35 Å channel formed by the tail and ejection proteins. These findings establish N4 as a distinctive model for protein translocation through biological channels.

## INTRODUCTION

Tailed bacteriophages, unlike most eukaryotic viruses, deliver their genetic material into the host bacterium while leaving the empty viral capsid outside. Various strategies are employed to translocate genomic material across the complex and diverse bacterial cell walls ^1^. Of particular interest are phages whose tail apparatus is too short to penetrate the 300 Å-thick cell envelope of Gram-negative bacteria. Formerly known as *Podoviridae*, these phages have a short, non-contractile tail that interrupts the capsid’s icosahedral symmetry ^2^ and provides a conduit for host attachment and genome ejection. The general ejection strategy of short-tailed phages involves encapsidating multiple copies of three specific proteins, known as ejection proteins ^3^, which are ejected into the host prior to DNA. In this process, the ejection proteins oligomerize and form a continuous channel that couples the DNA-filled capsid to the bacterial protoplasm, enabling successful infection and replication.

Phage T7, a prototypical *E. coli* phage and the classic model system for *Podoviruses*, has proven invaluable in deciphering the organization and function of ejection proteins. T7 stores three proteins, gp14, gp15, and gp16, in an 8:8:4 arrangement stacked above the portal ^4,5^. These proteins are released into *E. coli* to form a transenvelope channel, where gp14 creates the outer membrane channel (OMC), gp15 acts as a periplasmic tunnel (PT), and gp16, the largest subunit, forms a poorly understood inner membrane complex (IMC) that binds the bacterial membrane ^6–8^, while recruiting the host RNA polymerase ^9–11^. *In vitro* reconstituted complexes of PT gp15 bound to a fragment of gp16 have been reported ^7,12,13^, as well as an *in situ* analysis of T7 infecting *E. coli* mini cells proved essential to visualize large quaternary structure rearrangements in the phage tail, which are coupled to genome ejection ^14^. Collectively, the DNA ejectosome is a transient assembly that facilitates the delivery of T7 DNA (~39.9 kb) using the host RNA polymerase as a downstream motor ^1^, but must also somehow become inactive, or disassemble after genome ejection, to prevent permeabilizing the bacterial inner membrane ^6^.

While T7 has provided invaluable evidence in short-tailed phage ejection, it has become clear that there is a vast diversity of tail morphologies encompassed within this long-standing classification system. Specifically, the *E. coli* phage N4 and the growing number of N4-like bacteriophages differ significantly from small podoviruses like T7 both in genetics and structure. As such, N4-like phages have recently been reclassified into the *Schitoviridae* family ^15^. N4-like phages have a genome size of about 75 kb, nearly twice that of T7, and encode around 70–90 ORFs, including a characteristic large virion-associated RNA polymerase (vRNAP) unique to this family of bacterial viruses. Recently, we characterized the *Schitovirus* DEV that infects *Pseudomonas aeruginosa* and mapped an operon consisting of three ejection proteins, roughly analogous to T7 gp14, gp15, and gp16, despite lacking detectable sequence similarity ^16^. Cryo-EM analysis showed that DEV gp14-like OMC (gp73) and gp15-like PT (gp72) are entirely alpha-helical and form an aqueous channel about 25–35 Å wide, large enough to allow DNA passage. Additionally, we found that DEV gp14-like factor exhibits pore-forming activity *in vitro*, supporting its role as a channel at the OM. Interestingly, we found that the ejection operon of DEV lacks a clear gp16 analog; instead, the third ejection protein corresponds to the previously annotated vRNAP, a giant RNA polymerase ^3^, hallmark of the *Schitoviridae* family ^15^. This protein has been extensively studied in phage N4, particularly regarding its function and role during early transcription ^17^. The massive 3,500-residue vRNAP ^18,19^, present in 4 ± 1 copies inside the mature virion ^20^, is ejected into the host upon infection ^17^. This enzyme is responsible for an early burst of RNA synthesis observed immediately after N4 infection, even after inhibiting the host RNAP with rifampicin ^21^. Interestingly, N4 vRNAP efficiently transcribes only denatured N4 DNA *in vitro* but is inactive on native N4 DNA, suggesting the transcription of early promoters requires host DNA gyrases to introduce negative supercoils into the phage genome, leading to extrusion of the unique N4 single-stranded DNA (ssDNA) hairpin promoter ^17^. N4 vRNAP initiates infection by transcribing the early genes gp1 and gp2, as well as gp15 and gp16. gp1 and gp2 act as cofactors for the heterodimeric N4 RNAPII (gp15:gp16), which, in turn, is responsible for the transcription of the middle genes ^17^. Among these middle transcripts, N4SSB redirects the host RNAP to late promoters, mediating the expression of late genes involved in virion assembly, DNA replication, packaging, and host lysis.

**Figure 1:**
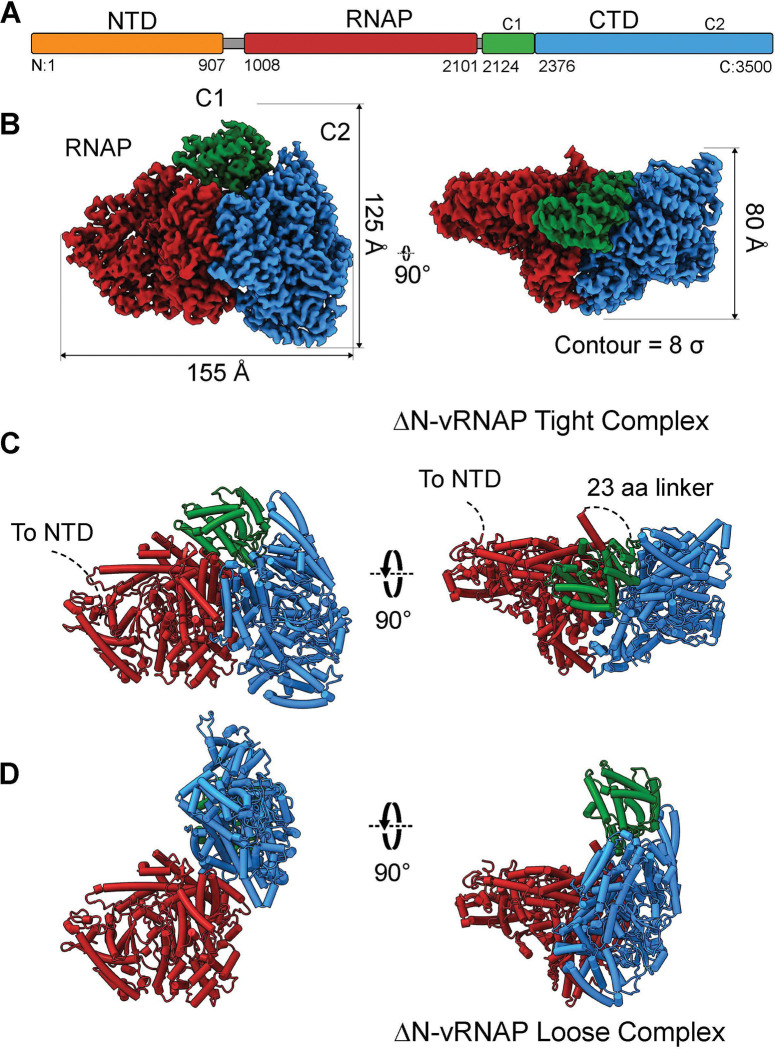
Cryo-EM reconstruction of N4 vRNAP. (**A**) Linear schematics of vRNAP domain organization. (**B**) Experimental density of ΔN-vRNAP tight complex colored by domain. (**C**) Ribbon diagram of ΔN-vRNAP tight complex colored by domain. (**D**) Ribbon diagram of ΔN-vRNAP loose complex.

In stark contrast to the N4 transcriptional program, which has been studied extensively ^17^, many key questions about the role of phage N4 vRNAP as an ejection protein and its part in the DNA ejectosome remain unanswered. vRNAP (gp50) consists of three predicted domains: a likely membrane-spanning N-terminal domain (NTD), a single-subunit RNA polymerase (RNAP) domain, and a large C-terminal domain of unknown function (CTD) (Fig. 1A). Of particular interest is how multiple copies of this 3,500-residue enzyme manage to pass through a narrow tail and become a functional RNA polymerase inside the cell. Surprisingly, N4 vRNAP was not visible in a medium-resolution asymmetric reconstruction of the mature N4 virion, and no clear loss of density was observed comparing the WT N4 with a mutant lacking the vRNAP ^20^. Recent high-resolution cryo-EM studies of N4 ^22^ and other *Schitoviruses*, such as the DEV ^16^ and the *Shigella* phages Moo19 and B2 ^23^, containing homologous vRNAP have also failed to visualize the structure of vRNAP in its native state. The only structural information about N4 vRNAP is limited to previous crystal structures of the transcriptionally active RNAP domain ^24^, which makes up the middle third of the full-length protein. The active RNAP domain is a divergent single-subunit RNA polymerase fold similar to T7 ^25^ and mitochondrial RNAP. The structure of the remaining two-thirds of this ejection protein remains completely unknown. Furthermore, it is unknown how such a large protein can be threaded through the narrow 30–45 Å tail of phage N4, given that just the solved crystal structure of the RNAP is nearly twice the diameter of the tail channel. This led to a model whereby the vRNAP is unfolded during ejection ^17^.

In this study, we use a complementary top-down and bottom-up approach by determining the cryo-EM structures of both the full-length recombinantly expressed vRNAP and the fully asymmetric N4 virion tail. These structures provide key insights into both the enzyme itself and the ejection process of vRNAP and other ejection proteins. We localize N4’s smaller ejection proteins, gp51 and gp52, inside the virion and demonstrate their function as pore-forming channels. Lastly, we uncover a novel and unexpected tail-gating complex that connects the N4 receptor-binding protein to the tail tube, its tail needle, and the release of ejection proteins. Overall, this study describes the specific mechanisms of ejection initiation for the prototypical *Schitovirus* N4.

## RESULTS

### Bottom-up analysis of N4 ejection protein gp50 (vRNAP)

Despite the large size of N4 vRNAP (gp50) and the presence of 4 ± 1 copies within the virions, attempts to visualize this giant protein inside the virion using cryo-EM and a genetic knockdown of gp50 have been unsuccessful ^20^. A recent high-resolution asymmetric reconstruction of the mature N4 virion tentatively placed vRNAP inside the tail apparatus ^22^, although the internal tail volume is not large enough to accommodate 4 ± 1 copies of a ~383 kDa enzyme (*see below*). We performed bubblegram imaging on intact N4 virions to probe the rough localization of vRNAP inside the capsid. Bubblegramming leverages the differential rate of damage of protein compared to nucleic acid under an electron beam. Proteinaceous material will decay first, resulting in characteristic bubbling due to the release of trapped gases inside the capsid. This method has been employed to gain a deeper understanding of several phage ejection proteins, including T7 and PhiKZ ^26–28^. For N4, we observed expected bubbling at the tail vertex corresponding to the portal as well as bubble clusters typically placed above the portal with distinct separation between the portal bubbling (**Supplementary Fig. 1A**). When measuring the distance from the tail:capsid interface to the bubble center across all virions, we observed a trimodal distribution (**Supplementary Fig. 1B**), with the first peak likely reflecting the portal and associated proteins, and the other two peaks suggesting proteinaceous material extending up to 600 Å inside the capsid. Thus, bubblegram imaging suggests a moderately stochastic placement of proteinaceous material likely belonging to 4 ± 1 copies of vRNAP inside the capsid with distinct loci of bubbling.

We used a bottom-up approach to decipher the architecture of N4 vRNAP (M.W. ~383 kDa). We purified the recombinant gp50 to homogeneity, which was then vitrified and subjected to cryo-EM data collection (**Supplementary Fig. 2A, B**). Single particle analysis (SPA) of cryomicrographs revealed a heterogeneous mix of particles with two predominant and two minor populations (**Supplementary Fig. 3**). The two major populations were reconstructed to 2.6 and 2.8 Å resolution, respectively (Table 1), allowing us to build atomic models for the isolated RNAP domain (res. 1007–2101) and a tightly bound complex of RNAP and CTD with an unresolved NTD, referred to as ΔNv-RNAP tight complex (res. 1007–3500) (Fig. 1B, C). N4 isolated RNAP and ΔN-vRNAP tight complex were real-space refined to a model to map correlation coefficients of CC = 0.90 and 0.84, respectively (Table 1, **Supplementary Fig. 4A, B**).

The minor populations present on the grid were more challenging to decipher (**Supplementary Fig. 3**). One population was likely consistent with the isolated CTD; however, due to its preferred orientation, we were unable to compute a meaningful 3D reconstruction. The other smaller population represents a distinct quaternary structure of RNAP and CTD referred to as ΔN-vRNAP loose complex (Fig. 1D). We were able to resolve a 4.3 Å map of this alternate quaternary arrangement of ΔN-vRNAP with a small 18,000 particle subset of a much larger stack consisting of ~325,000 particles putatively in this loose state (**Supplementary Fig. 4C**). This reconstruction allowed us to dock, and rigid-body refine RNAP and CTD, which revealed CTD rotates relative to RNAP by 100°, compared to the ΔN-vRNAP tight complex (Fig. 1C). Difficulty aligning all ~325,000 particles belonging to this state suggests that the resolved loose complex map using ~18,000 particles may be one stable state on a continuum of motion of the CTD with respect to the RNAP domain. No 2D classes representing NTD (Fig. 1A), either alone or as part of vRNAP, could be resolved. This is likely explained by a 100-amino-acid low-complexity sequence connecting the NTD and RNAP (res. 907–1006) that connects the two folded domains, suggesting a disconnected NTD complex. We observe no evidence of degradation, suggesting that the NTD is present on the grid; however, both 2D and 3D reconstruction may be hindered by its lack of ordered structure or by smaller subdomains that are easily masked by the stronger signals from the heterogeneous mix of RNAP and CTD.

### Quaternary organization of RNAP and C-terminal domains

The extreme conformational heterogeneity captured by SPA prompted us to investigate the relative arrangement of RNAP and CTD tight complex. In the isolated RNAP and the tight complex, the overall organization of the RNAP is similar to crystal structures previously reported, with significant local variations likely caused by crystal contacts and crystallization conditions (RMSD with PDB:2PO4 ~ 4.7 Å and 2.0 Å for isolated RNAP and tight complex RNAP, respectively) ^24^. In contrast, the structure of N4 CTD was previously unknown. vRNAP CTD is a large structure, quite wide in two dimensions (125 Å × 100 Å) and very narrow in the third (50 Å). The domain is roughly divided into two subdomains: C1 (res. 2124–2375, Fig. 1A–D green) and C2 (res. 2376–3500, Fig. 1A–D blue). The C1 domain possesses a conserved HExxH motif, found in zincin metalloproteases and radical S-adenosylmethionine (radical SAM) enzymes ^29^. C2 is large (~110 kDa), mostly α-helical, and shows no significant structural homology to any deposited protein structures or even AlphaFold predictions of biochemically well-studied proteins lacking solved structures.

**Figure 2:**
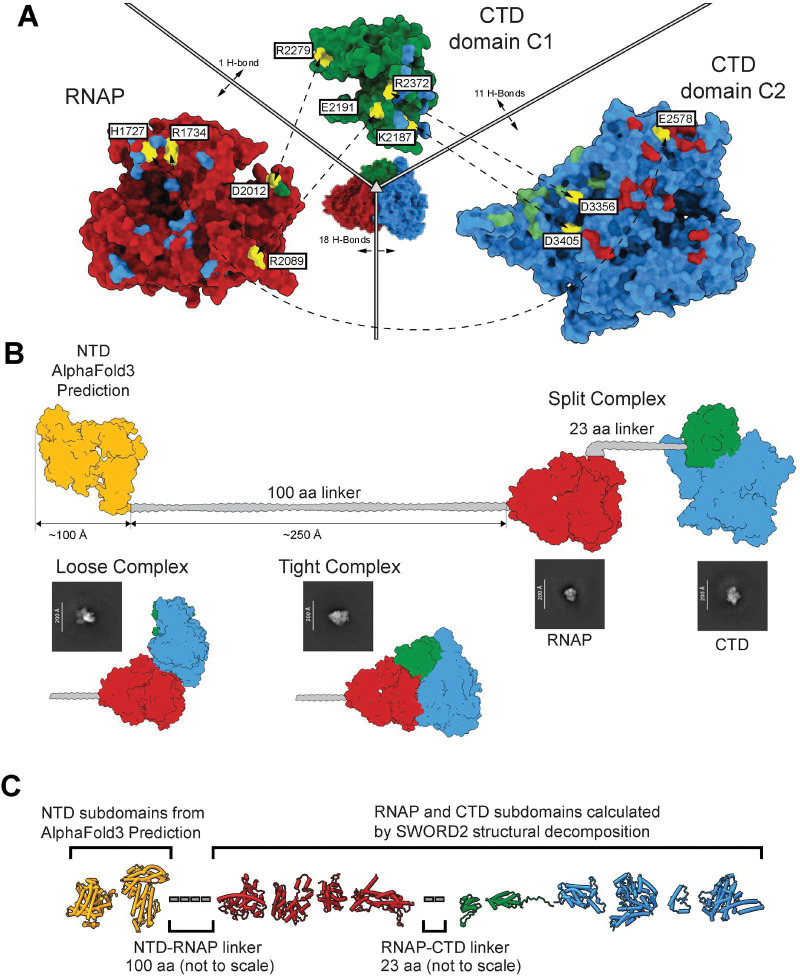
Plastic quaternary organization of vRNAP domains. (**A**) Map of binding interfaces and interactions that stabilize vRNAP tight conformation. Yellow indicates salt bridges with the corresponding residue linked by a dashed line. Other colored residues indicate hydrogen bonding residues to subunits of the same color. (**B**) General schematics of all putative domain organizations of vRNAP observed on grid with supporting 2D classes. (**C**) Putative model of beads-on-a-string pre-ejection conformation. NTD subdomains were predicted by AlphaFold3; RNAP and CTD subdomains were predicted by the protein peeling structural decomposition algorithm SWORD2.

The tight complex of the RNAP (Fig. 1B,C) and CTD forms a compact structure where the CTD binds the RNAP on the opposing face of the DNA hairpin promoter binding site ^24^, leaving the region open for promoter binding. Despite a large binding interface of 1,659 Å^2^, PISA analysis of the C2 and RNAP interface indicates a weak interaction with a ΔG of nearly zero at −0.2. Interestingly, interactions are slightly stronger at a ΔG of Ȓ2.1 for RNAP to C1 with a 725 Å^2^ interface. A salt bridge between D2012 (RNAP) and R2279 (C1) (Fig. 2A) likely serves as a clasp for stabilizing the tight complex, which is not predicted to be stable in solution. Interactions between the C1 and C2 are slightly stronger, although we observe contiguous density connecting them, and the designation as separate domains is more a result of the fold than the expected dynamics.

We next focused on the intramolecular linkers connecting vRNAP domains. As described above, a ~100-residue linker between NTD and RNAP (res. 907–1007) could span as much as 250 Å, explaining the challenge of aligning this domain with the rest of vRNAP during SPA. AlphaFold3 predicts the NTD as two globular helical subdomains, and putative membrane-spanning α-helices (MSH) are predicted between residues 224 and 278 (**Supplementary Fig. 5A**). This prediction was validated *in vitro,* where the isolated gp50-NTD can be incorporated into membrane nanodiscs (**Supplementary Fig. 5B, C**), consistent with vRNAP membrane localization during infection ^30^. Similarly, a 23-amino-acid linker, spanning residues 2102–2123, connects RNAP to the CTD and is invisible in our structure. Thus, vRNAP can be rationalized as three globular, folded units flexibly connected by at least two extended linkers. These linkers may explain why SPA of vitrified vRNAP also captured individual RNAP and CTD domains if the two domains are separate in a “split complex”. All possible resolved maps and 2D classes are explained by these three putative states (Fig. 2B).

The globular structures of both RNAP and CTD do not provide any clarification on how the entire assembly manages to squeeze through the ~35 Å diameter of the tail-tube ^22^. We used SWORD2 ^31^ to perform structural decomposition of ΔN-vRNAP and to begin unraveling how this enzyme may unfold and refold into a fully functional enzyme upon translocation through a narrow channel across the double-membrane *E. coli* cell wall. SWORD readily decomposed RNAP and CTD into nine subdomains (Fig. 2C) with a high likelihood score. This peeled topology, along with the two globular subdomains predicted by AlphaFold3 in the NTD, results in a total of eleven roughly globular and flexibly linked subdomains, resembling beads-on-a-string (Fig. 2C).

### A bulky CTD autoinhibits RNA polymerase activity

**Figure 3:**
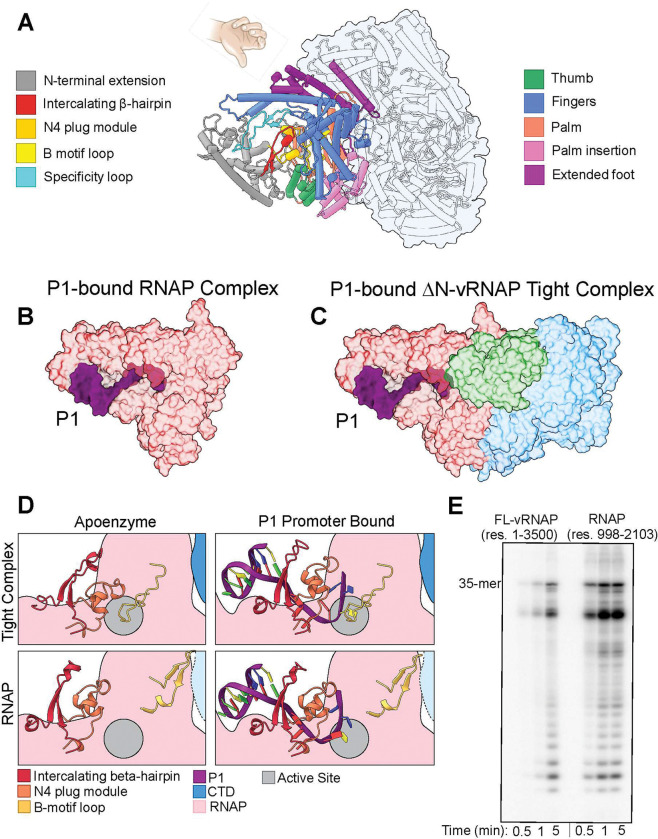
CTD allosterically regulates vRNAP transcriptional activity. (**A**) ΔN-vRNAP tight complex atomic model with RNAP colored by subdomains. (**B**) Surface model of the tight complex with the DNA hairpin P1 promoter bound. (**C**) Surface model of isolated RNAP with P1 promoter bound. (**D**) Conformational changes in RNAP subdomains due to P1 and CTD binding. (**E**) Radiolabeled transcription runoff assay of FL-vRNAP (res. 1–3500) and recombinant mini-vRNAP (res. 998–2103) shows less processivity for full-length protein compared to mini-vRNAP.

N4 RNAP domain adopts a typical single subunit RNAP (ssRNAP) fold that resembles a right hand with three subdomains: fingers, palm, and thumb (Fig. 3A). The CTD buries a large surface area of the RNAP occupied by the fingers and palm including the palm insertion and extended foot domains, but despite the extensive interface, the overall structure of the N4 RNAP domain is relatively unchanged in complex with the CTD (RMSD 3.7 Å) (Fig. 3A), barring a few changes in subdomains unique to N4 and a subtle shift of the fingers.

N4 vRNAP is highly specific for a ssDNA template and is transcriptionally active on such a template ^25^. To assess if the ssDNA template induces conformational changes in vRNAP, we also performed cryo-EM SPA on vRNAP bound to a single-stranded P1 promoter (Fig. 3B, C). Initial 2D classes were nearly identical to the apo structure and again resolved two populations of the isolated RNAP and ΔN-vRNAP tight complex bound to P1 that we refined to 2.8 and 2.9 Å resolution, respectively (Table 1, **Supplementary Fig. 4D, E**). Similar to the apoenzyme, two populations of CTD alone and the loose complex were visible in 2D classes but unresolved in 3D. In both structures, the P1 promoter inserts deeply into the RNAP active site, and the DNA hairpin binds as expected (**Supplementary Fig. 6A, B**) based on previous crystallographic studies ^32^. The isolated RNAP makes 10 hydrogen bonds (H-bonds) and 130 van der Waals contacts with P1. In contrast, RNAP in the tight complex makes 12 H-bonds and a total of 126 van der Waals contacts (**Supplementary Fig. 6C**). Surprisingly, the RNAP and CTD binding interface was unchanged in our apo and P1-bound structures, suggesting that the promoter’s binding is insufficient for the release of CTD from RNAP. Even if the RNAP is globally unchanged in all observed structures, the localization of CTD (either with or without P1) results in allosteric changes in three crucial motifs of the RNAP core: the intercalating β-hairpin, the B-motif loop, and the N4 plug module (Fig. 3D). The intercalating β-hairpin is a conserved domain in ssRNAPs, which helps unwind dsDNA ^33^. The B-motif is a conserved motif in ssRNAPs as well and is responsible for the coordination of incoming NTPs; however, in N4, it is unique in that a portion of the motif exists on a dynamic loop, the B-motif loop ^24^. The N4 plug module is entirely unique to N4 vRNAP and is suggested to inhibit polymerase activity by occupying the active site in concert with the B-motif loop ^24^.

Cryo-EM reconstructions of the four high-resolution states (Fig. 3D) provide clear evidence that the CTD drives the B-motif loop into the active site in a conformation that we predict is inactive. Without the CTD, the B-motif loop is folded into a beta hairpin that sits outside the active site. Interestingly, there is no direct contact between the CTD and B-motif loop, indicating that the hairpin loop is moved and released via an allosteric mechanism. Finally, in the P1-bound tight complex, the P1 promoter lifts the intercalating β-hairpin and the N4 plug module, but the B-motif loop still occupies the polymerase active site, suggesting that promoter binding is insufficient to release the B-motif loop and CTD. The P1 promoter is relatively unchanged in overall structure when comparing the isolated RNAP and the tight complex. A few contacts are added or gained due to a more relaxed structure in the isolated RNAP. The only major difference is that the −1 nucleotide is flipped away from the active site in the tight complex (**Supplementary Fig. 6B**), likely due to steric hindrance of the B-motif loop conformation that occupies the active site in the tight complex conformation.

To experimentally determine the role of the C-terminal domain on transcription, we compared the enzymatic properties of full-length vRNAP and the recombinantly expressed isolated RNAP domain (res. 998–2103) ^25^. We performed radiolabeled runoff transcriptional assays using both the full-length (FL) vRNAP and the RNAP domain alone on an 81-nt N4 native sequence ssDNA template, which contained the P1 hairpin promoter (Fig. 3E). We observed activity for both polymerases, showing slow overall transcription for both. However, the FL enzyme synthesized less RNA product as a function of time, consistent with the proposed autoinhibitory effect of the CTD. Finally, while not resolved to a high enough resolution to compare directly, the overall placement of the domains in the loose complex suggests that this conformation exists in the active state.

### Top-down analysis of N4 ejection proteins gp51 and gp52

**Figure 4:**
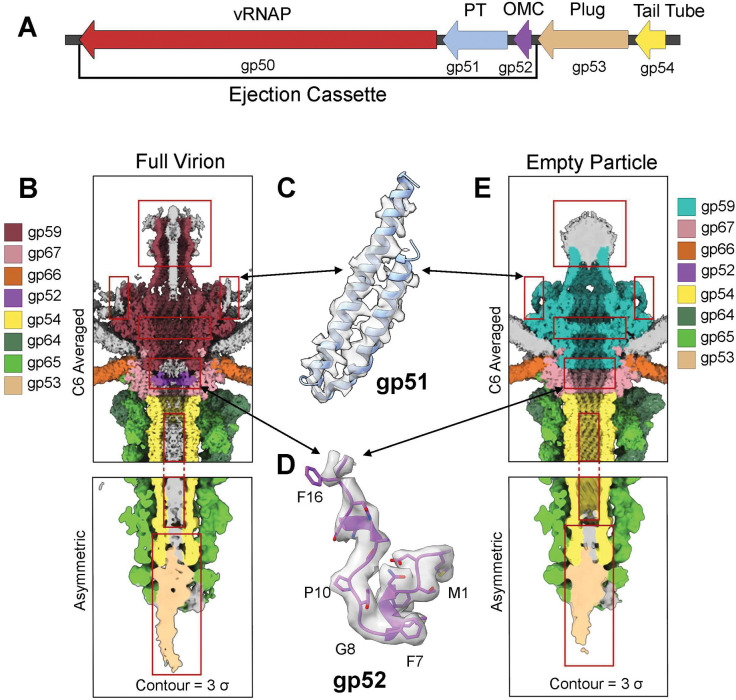
Asymmetric cryo-EM reconstructions of N4 full virion and empty particle identify ejection proteins gp51 and gp52. (**A**) Linear schematic of genomic organization of the N4 ejection cassette (gp50-gp51-gp52) with adjacent ejection-related proteins (gp53-gp54). (**B**) Cross-section of full virion density with major differences from empty particles boxed in red. (**C**) Isolated density of α-helical hairpin surrounding the portal is assigned to gp51. (**D**) Isolated density of gp52 fragment (res. 1–16) localized to the tail lumen. (**E**) Cross-section of empty particle density with major differences from full virions boxed in red.

N4 vRNAP (gp50) is part of a conserved *Schitoviridae* operon that encodes three ejection proteins: gp50, gp51, and gp52 (Fig. 4A). We took a top-down approach to identify the other two putative subunits, gp51 (644 aa) and gp52 (150 aa), which are homologous to phage DEV gp73 and gp72, respectively ^16^. We purified N4 virions to homogeneity (**Supplementary Fig. 7A, B**) and determined the high-resolution structure of phage N4 tails from filled virions (FV) and empty particles (EP) to 3.2 Å and 3.6 Å, respectively (Fig. 4B, E, **Supplementary Fig. 8A-E**, Table 2). The tail has a total vertical length of 450 Å, formed by the HT-adapter (gp67), tail tube (gp54), and tail plug (gp53), and is attached to the head through interactions between the HT-adapter and the portal (gp59). Surrounding the tail is a six-fold symmetric non-contractile sheath formed from a heterodimer of the minor subunit gp64 and the major subunit gp65 ^22^.

Tails from EPs are remarkably similar in structure to FVs, and comparison of the similarly resolved maps (Fig. 4B, E) identified three critical differences after the genome is ejected from the capsid. First, the portal undergoes two major changes: the collapse of the barrel helices beyond residue 667 ^20^ (**Supplementary Fig. 9A**) and the narrowing of the portal tunnel from 33 Å to 25 Å due to restructuring of the tunnel loop (**Supplementary Fig. 9B, C**). Second, the ejection protein gp51, which forms a α-helical hairpin adjacent to the wingtip domain of the portal, disappears in the empty virions (Fig. 4C, **Supplementary Fig. 9A**). In phage DEV ^16^, this protein folds into a nonameric tube after ejection, functionally analogous to phage T7 PT ^7,13^. Third, we identified 12 fragments of approximately 16 residues inside the lumen of the HT-adaptor, which are absent in EP (Fig. 4D), comprising the N-terminus of the ejection protein gp52 ^16^. The full-length gp52 consists of 150 residues, and the remainder of the protein likely orients downwards in the tail, where a recent asymmetric reconstruction of the N4 virion also identified spurious density ^22^.

**Figure 5:**
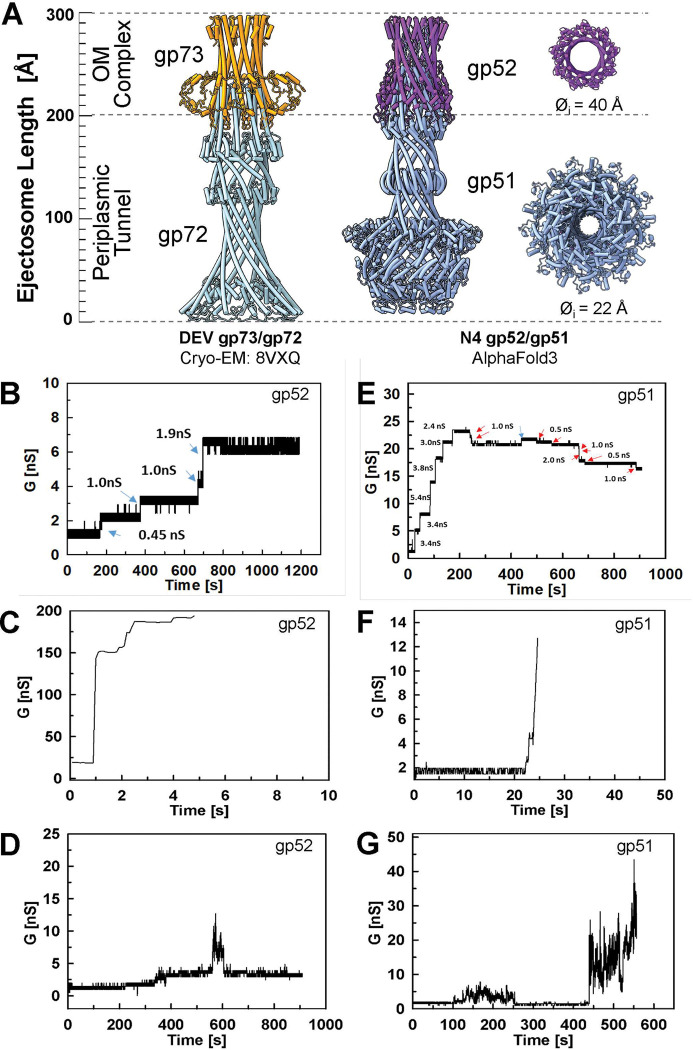
Channel activity of N4 ejection proteins gp51 and gp52. (**A**) Comparison of the cryo-EM structure of the nonameric *Pseudomonas* DEV phage gp73/gp72 ejection channel with the AlphaFold3 prediction of the homologous gp52 and gp51 proteins of the *Escherichia coli* N4 phage. The outer membrane complex proteins (OMC) are gp73 and gp52. The inner diameters (Ø_i_) of the predicted pores of gp52 and gp51 are indicated. (**B-G**) Lipid bilayer experiments were performed using diphytanoyl phosphatidylcholine (DphPC) membranes bathed in 10 mM HEPES pH 7.4, 1 M KCl as an electrolyte, and at −10 mV applied potential. All samples were added to both sides of the cuvette. After formation of the membrane across the aperture, each experiment was recorded for 15 minutes or less in the event of membrane rupture. Blue arrows indicate open channels, and red arrows indicate channel closure events. Current traces of gp52 in DDM buffer. Channel-forming activity was observed at protein concentrations of 8 ng mL^−1^ (**B**), 10 ng mL^−1^ (**C**), and 4 ng mL^−1^ (**D**). Current traces of gp51 in DDM buffer. Channel-forming activity was observed at protein concentrations of 320 ng mL^−1^ (**E**), 80 ng mL^−1^ (**F**), and 240 ng mL^−1^ (**G**).

Using our knowledge of the recombinantly expressed DEV ejection channel formed by 9 copies of gp72 and 9 copies of gp73 ^16^, we used AlphaFold3 to predict gp51 and gp52 with the same stoichiometry. With moderate confidence scores, the predicted N4 gp51 and gp52 structure formed a hetero-nonameric trumpet-shaped complex ~300 Å in length with an internal diameter ranging from ~20 Å to 40 Å (Fig. 5A), which is stabilized by extensive protein-protein interactions at their N-termini. This prediction is remarkably similar to the cryo-EM reconstruction of phage DEV ejection proteins gp72:gp73 despite the negligible sequence identity (>10%) between DEV and N4 ejection proteins (Fig. 5A). The AlphaFold3 model of gp52 suggests the protein forms a putative channel with an internal diameter of 40 Å primarily composed through the nonameric arrangement of an amphipathic α-helix (res. 69–131) and a hydrophobic moiety (res. 1–68) (**Supplementary Fig. 10A**). Similarly, the AlphaFold3 model of gp51 suggests an elongated tunnel-like channel, putatively the PT, about 140 Å in length, that generates a 30 Å wide tunnel (Fig. 5A). MemBrain ^34^ also detected putative membrane spanning helices at the N- and C-termini of N4 gp51 (**Supplementary Fig. 10B**). We purified recombinant gp52 and gp51, and consistent with this prediction they behave like membrane proteins in solution and were finally solubilized using 0.05% n-dodecyl-D-maltoside (DDM).

### Gp51 and gp52 integrate into lipid membranes and form membrane-spanning channels

Phage ejectosomes need to provide a continuous channel from the phage head to the cytoplasm of the infected bacterium. Previously, we showed that the *Pseudomonas* phage DEV utilizes the gp73 and gp72 proteins as OMC and PT proteins, respectively ^16^. AlphaFold3 predictions indicate that phage N4 gp51 and gp52 proteins form a complex and might fulfill similar roles in DNA ejection by the N4 phage across the cell envelope of *E. coli* (Fig. 5A). To test this hypothesis, we performed lipid bilayer experiments to determine whether gp52 forms membrane-spanning channels similar to DEV gp73. To exclude contamination with endogenous porins of *E. coli*, gp51 and gp52 were purified from the *E. coli* (DE3) omp8 strain (**Supplementary Fig. 11A**), which lacks all major pore-forming proteins OmpF, OmpC, LamB and OmpA ^35^. Control experiments with only electrolyte or when protein buffer containing DDM was added to the lipid membrane did not show any interaction with the membrane or pore activity (**Supplementary Fig. 11B, C**). By contrast, the addition of the purified gp52 protein to lipid membranes resulted in current increases indicative of pore formation (Fig. 5B–D). Interestingly, only occasionally did we observe a steady stepwise current increase (Fig. 5B, **Supplementary Fig. 12A, B, C**), which is typical of bacterial porins ^36^ or pore-forming toxins ^37^. These gp52 pores were prone to channel closures as indicated by the downward spikes (Fig. 5B). We also observed events of a sudden, large increase in membrane currents, which ruptured the membrane (Fig. 5C, **Supplementary Fig. 12C, D, E**). In many instances, we recorded unstable pores or membrane disturbances as indicated by rapidly fluctuating currents (Fig. 5D). Overall, we tested gp52 in 24 individual lipid membranes. These experiments demonstrate that gp52 interacts with membranes and forms membrane-spanning channels with channel conductance ranging from 0.45 nS to 1.9 nS.

We also examined whether the periplasmic tunnel protein gp51 (Fig. 5A) interacts with membranes. Surprisingly, lipid bilayer experiments revealed that the purified gp51 protein (**Supplementary Fig. 12A**) has channel-forming activity in contrast to its DEV counterpart, gp72 ^16^. Similar to N4 gp52, we observed stable gp51 pores with conductances ranging from 0.5 to over 5 nS (Fig. 5E, **Supplementary Fig. 13A-C**). We also observed rapid and large insertions that frequently led to membrane rupture (Fig. 5F, G, and **Supplementary Fig. 13D, F**), as well as flickering and noisy insertions (Fig. 5G, **Supplementary Fig. 13A-F**). Overall, we tested 18 individual membranes with the purified gp51 protein and observed frequent interactions with lipid bilayers and heterogeneous populations of channels. This is in contrast to the equivalent periplasmic tunnel protein gp72 of the DEV phage ^16^, indicating differences in their capacity to interact with membranes, probably due to the presence of hydrophobic α-helices (**Supplementary Fig. 10B**), which are not found in DEV gp72. Notably, the gp51 concentrations needed to observe channel activity were at least 10 times higher than those of gp52, probably reflecting the different propensities of the individual proteins to insert into membranes. Overall, our data show that the phage N4 gp52 and gp51 proteins form heterogeneous channels in lipid membranes with widely varying physical properties, which differ from those of bacterial pore-forming outer membrane proteins that typically create stable channels in lipid bilayer experiments.

### A tail-gating complex couples receptor binding to ejection protein release

**Figure 6:**
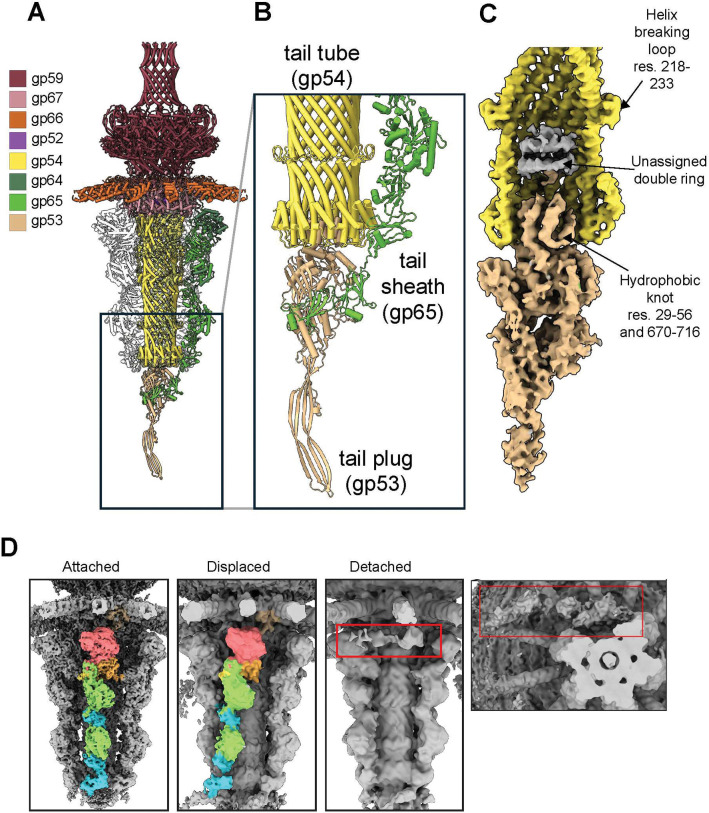
Structure of the N4 asymmetric tail-gating complex. (**A**) Ribbon diagram of asymmetric N4 tail with all sheath heterodimers (gp64-gp65) shown in light gray except for the gp53 interacting sheath heterodimer, which forms the tail-gating complex. (**B**) Atomic model of asymmetric tail-gating complex. (**C**) Cross-section of experimental density colored by component showing unique internal features. Density for sheaths and front tail-tube protomers has been removed. (**D**) Densities showing the different conformations of the N4 sheath. Detached conformation shown with an additional viewpoint to show weak density of the sheath (boxed in red).

The tail of phage N4, in both FV and EP, is plugged by a large protein with a globular core and a long, feather-shaped β-sheet protrusion that we identified as gp53 (Fig. 6A, B). A focused reconstruction of the tail plug hub (Fig. 6C, **Supplementary Fig. 14A**) revealed a convincing density that we interpreted using an AlphaFold model of gp53. The plug identity is further supported by gp53’s presence in our LC-MS analysis (**Table S1**). The majority of gp53 sits outside of the tail-tube, and a single assembly of the non-contractile sheath (gp64:gp65) protein binds the globular core of gp53. In N4, the non-contractile sheath is formed by a heterodimer of gp65 and gp64 (**Supplementary Fig. 14B**). Long known to be the N4 receptor binding protein ^38^, gp65 has a modular organization with gp64 consisting of 10 domains (D1-D10), and in the focused reconstruction of the tail-gating complex, gp65 tightly binds the core of gp53 through the final 200 residues, which comprise domains 9 and 10 (Fig. 6B).

In our focused asymmetric map, an additional sheath is somewhat stabilized through differential binding of D9 and D10 (**Supplementary Fig. 14A**), while the remaining four show minimal interaction in our map. A non-contiguous stretch of gp53 residues (29–56 and 670–716) forms a hydrophobic knot (Fig. 6C) comprising a complex weaving of six short-alpha helices that corks the tail-tube base. Density continues for a few residues beyond this knot before the remaining 160 residues are lost in the tail lumen. A poorly resolved double ring structure is observed (Fig. 6C), perhaps belonging to the remaining gp53 residues, multiple copies of ejection protein gp52, or a combination thereof. Interestingly, the tail tube appeared sealed in both the full and empty particles. The unique interaction between gp65 and gp53 could allow the plug protein to remain in proximity and rebind the tail base after DNA ejection, facilitated by the hydrophobic knot, explaining this phenomenon at least *in vitro*.

Further supporting the asymmetric interactions of the N4 non-contractile sheath, 3D classification using a focused mask on a single sheath assembly revealed two divergent classes (Fig. 6D, Table 2). One showed the protomer slightly displaced, with D8 detached from the tail tube and bound to a neighboring protomer. In the other, the protomer was absent, but inspection at low contour revealed weak density away from the tail core, which would require a 90 ° rotation about the HT-adapter anchored gp65 C-terminus. This low-contour density likely results from a subset of particles in the final particle stack, with other particles having freely moving and unanchored sheath assemblies. This suggests that while one or two sheath proteins may be tightly anchored to the tail core by gp53, the other four might be free to detach and interact with the polysaccharide-rich environment of the extracellular space.

## DISCUSSION

Ejection proteins, which are essential for infectivity and encoded by most *podoviruses*, exemplify the concept of conformational plasticity and structural adaptability perhaps better than any other protein in nature ^3^. These encapsidated proteins undergo extreme conformational and oligomerization changes during expulsion through the phage tail, leading to the formation of a megadalton-sized DNA-ejectosome that spans the cell envelope of Gram-negative bacteria. The DNA-ejectosome is not permanently open; otherwise, it would harm the host, so it must regulate its gate or potentially disassemble after the genome is ejected. Most of what is known about ejection proteins is inferred from studies in the model system T7, which was the first and remains the best-characterized phage for studying genome ejection. Nonetheless, T7 is much simpler than phage N4, which is the prototype of a rapidly expanding and surprisingly abundant family of phages recently renamed *Schitoviridae* ^39^. Not only is the N4 genome nearly twice the size of T7’s, but N4’s largest ejection protein, analogous to gp16 in T7, is a 3,500-residue RNAP ejected from the capsid, which has been studied as a unique RNAP for over 50 years ^17^. N4 requires three RNAPs—two encoded by the phage and one from the host.

In this study, we used a combination of top-down and bottom-up approaches to elucidate the structure and activity of the phage N4 ejection proteins as well as how phage attachment to a primary receptor triggers the ejection of these proteins into the bacterium. We made three discoveries.

**Figure 7:**
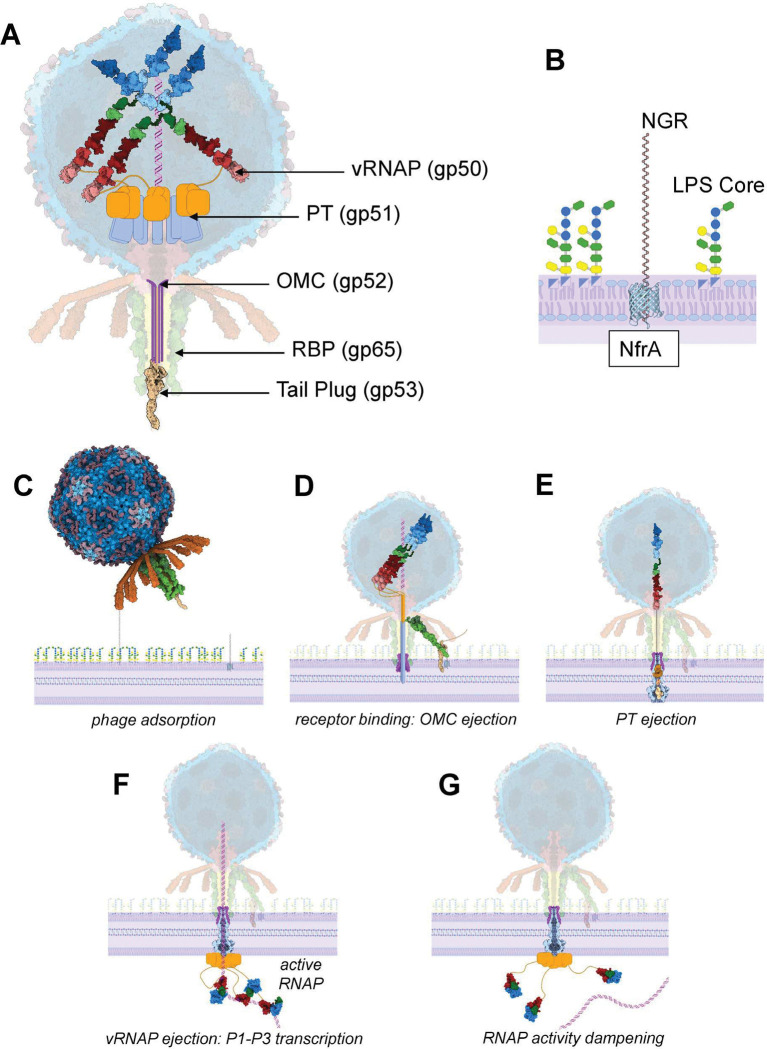
Composite model of N4 ejection protein-facilitated genome delivery. (**A**) Diagram of the infectious N4 virion with ejection proteins gp50, gp51, and gp52 poised for expulsion. In pre-ejection conformation, gp52 localizes inside the tail, gp51 surrounds the portal, gp50-NTD associates with gp51, and gp50 RNAP-CTD folds as beads-on-a-string inside the capsid. (**B**) Schematic diagram of phage N4 receptors at the E. coli surface: the novel glycan receptor (NGR), an exopolysaccharide exported by the proteinaceous receptor NfrA. (**C**) N4 appendages (gp66) reversibly bind NGR. (**D**) Irreversible binding of the RBP gp65 with the receptor NfrA, which displaces the plug (gp53), letting several luminal copies of gp52 to implant into the membrane as the OMC. (**E**) The ejection cascade continues, with gp51 being expelled through the tail and OMC to form a tunnel through the periplasm. (**F**) Gp50 follows in ejection through the interaction between gp51 and gp50-NTD. The inner membrane complex (IMC) is likely formed by both the gp51 C-terminus and the gp50-NTD. gp50-RNAP and CTD refold within the bacterial cytoplasm but stay anchored to the bacterial membrane by the linker. (**G**) Once transcription of early promoters is complete, the autoinhibition of transcription by gp50-CTD prevents unnecessary transcription from happening.

First, cryo-EM analysis of recombinant vRNAP establishes this protein as a novel single-chain multi-subunit RNAP, much more complex than phage or mitochondrial ssRNAPs, yet simpler than multi-subunit eukaryotic RNAP. This three-domain protein comprises a lipid-binding NTD loosely associated with the catalytic RNAP, which, in turn, neighbors a massive CTD of new fold. Single-particle analysis identified both a tight state of RNAP and CTD, as well as individual domains, suggesting a loose quaternary structure that was not affected by promoter binding. Although the CTD makes sparse contacts with RNAP, it allosterically regulates three crucial motifs of the RNAP core: the intercalating beta-hairpin, the B-motif loop, and the N4 plug module, thereby repressing transcriptional activity. *In vitro* transcription assays confirmed this inhibitory role of the CTD, which we speculate can only be exerted when the two domains are in direct contact. Computational decomposition analysis provides evidence for beads-on-a-string architecture characterized by as many as eleven globular sub-domains of approximately 250–300 amino acids, each large enough to fit through the phage tail channel. We propose that this topology represents the pre-ejection conformation of N4 vRNAP inside the N4 capsid (Fig. 7A), which is ejected into the host cell envelope, where it assembles and refolds.

Second, by combining bottom-up analysis of the isolated vRNAP and top-down studies of N4 virions, we discovered that the other two ejection proteins, gp52 and gp51, which are part of the same operon as vRNAP, are located in the pre-ejection conformation inside the tail and around the portal protein crown, respectively (Fig. 7A). After ejection, these proteins form an OM channel, like phage T7 gp14 ^4,5^, and a PT tunnel going from the OM to the IM, like T7 gp15 ^7,13^, which are first ejected during infection. This finding reconciles previous work that proposed the ejection proteins reside in the tail of phage 22 ^40^, while others found evidence of capsid localization, loosely bound to the portal barrel ^26^.

Third, through focused reconstructions and mass spec analysis, we deciphered the unexpected structure of the N4 tail-gating complex, which is characterized by a unique 12:6:1 symmetry mismatch. We found that asymmetry in the receptor-binding sheath subunit gp65 generates a 3-way signaling platform formed by tail plug (gp53), the receptor-binding factor (RBP, or sheath, gp65), and the ejection proteins residing inside the tail (gp52) and capsid (gp51 and gp50). We propose that the tail-gating complex links phage binding to the bacterial receptor, which could be either NfrA or NGR (Fig. 7B), or a combination of both ^38,41,42^, to a coordinated and synchronized release of ejection proteins into the bacterial cell envelope. Surprisingly, both the N4 mature virion and empty particle have a density for the tail plug, suggesting that the plug is not lost in the environment after receptor binding but remains tethered to the tail complex, available for reattachment after ejection proteins and DNA have been released. Although our cryo-EM data may not accurately represent an *in vivo* infection because empty N4 particles have released the DNA into a test tube rather than ejecting it into a cell via receptor binding, lateral displacement of the plug protein is indeed a common feature among *Siphophages* ^43^.

Based on the experimental evidence presented in this paper and a substantial body of data in the N4 literature, we propose the following model to describe the steps of N4 infection. The first step (Fig. 7C) is the nonspecific reversible adsorption of N4 to the host, which likely involves the interaction of 12 N4 appendages (gp66) with an exopolysaccharide, likely NGR, a polysaccharide putatively exported by N4’s secondary receptor NfrA ^38,42^. This step is conceptually similar to that of phages T4 and T7 ^44^ and facilitates the phage association with a specific receptor, NfrA or its exported polysaccharide NGR, which functions as a specific receptor ^38,42^. Once the phage gets closer to the outer membrane (Fig. 7D), the tail-gating complex is disrupted through interactions with the receptor NfrA or its exported polysaccharide NGR ^38,42^, which results in the lateral displacement and the dislodgment of the tail-gating complex, including the plug C-terminus. Displacement of these residues could act as a ripcord for tail luminal copies of gp52, serving as the initiator for ejection and insertion of the OMC into the outer membrane. The ejection cascade (Fig. 7E), initially powered by the pressurized genome stored inside the capsid, continues with the expulsion of gp51, which forms the PT spanning from OM to IM. In N4, the last C-terminal residues of gp51 are predicted to be hydrophobic and may form the actual channel crossing the IM. Together, the OMC and PT form the structural channel spanning the bacterial cell envelope, which we envision as a nonamer based on the stoichiometry observed in DEV ^16^. Peptidoglycan hydrolytic activity is likely required to penetrate the PG layer, although N4 ejection proteins do not appear to harbor this catalytic activity, which is presumably provided *in trans* by some other factors. vRNAP is the last ejection protein expelled from the capsid (Fig. 7F). We propose that this large protein exists in the beads-on-a-string architecture illustrated in Figure 2C, possibly maintained in a molten globule state inside the capsid due to the pressurized genome ^45^. vRNAP is ejected through the newly formed OMC:PT channel spanning the entire cell envelope and remains part of the DNA-ejectosome via the hydrophobic interactions made by its NTD and the C-terminus of gp51. We envision three copies of vRNAP associating with the nonameric PT, based on the observation that vRNAP is present in 4 ± 1 copies inside the capsid ^20^. In the host cytoplasm, without the pressure from the genome in the capsid ^46^, the subdomains likely refold to generate a compacted and catalytically active RNAP and CTD. This transcriptionally active state may allow the protein to promote DNA-dependent transcription of the first three promoters, pulling the genome into the bacterium. We speculate that each promoter (P1-P3) interacts with a different copy of the RNAP, which is specifically active when the single-stranded promoter hairpin associates with the RNAP. The specific role of CTD in early transcription remains unknown. Given its large size, which is conserved throughout *Schitoviruses* ^16^, it likely works alongside RNAP during early gene transcription, possibly by binding to or unwinding DNA. After N4 second RNAP (RNAP II) has been translated, and the phage moves into middle transcription, intramolecular closing of gp50 RNAP and CTD (Fig. 7G) would dampen transcriptional activity due to the intermolecular inhibitor effect of the latter, reconciling the observed function of the C-terminus as autoinhibitory of transcriptional activity.

In summary, this study provides a framework for understanding the structure, activity, and plasticity of the giant and mysterious vRNAP, which is ejected by phage N4 and likely all *Schitoviridae* into Gram-negative bacteria. This polymerase is not only the first single-chain, multi-domain RNAP identified to date, but it also exemplifies a remarkable case of phage adaptation to its host. Future research will need to determine how vRNAP unfolds during ejection and whether the transcriptional activity of vRNAP is also part of the genome ejection motor that enables *Schitoviridae* to deliver their genomes into Gram-negative bacteria.

## METHODS

### N4 phage preparation for cryo-EM

A 100 mL culture of *E. coli* MG1655 (GenBank: U00096.3) in LB was grown at 37°C up to OD_600_ = 0.2 (about 7.5 × 10^7^ CFU mL^−1^) and infected with N4 (GenBank: EF056009.1) at a multiplicity of infection (MOI) of 3. After 3 hours (h) of incubation at 37 °C with shaking, chloroform (0.5 mL) was added, and the incubation continued for 15 minutes (min). The lysate was centrifuged at 5,000 × g for 15 min, and the supernatant was recovered and filtered through a 0.45 μm filter. 58 g L^−1^ NaCl and 105 g L^−1^ polyethylene glycol (PEG) MW 6,000 were dissolved in the supernatant. The solution was incubated for 16 h at 4°C before pelleting the phage particles by centrifugation at 20,000 × g at 4 °C for 30 min. The pellets were resuspended in TM buffer (10 mM Tris-HCl pH 8, 10 mM MgCl_2_) and centrifuged on a CsCl_2_ 1.3 to 1.6 g cc^−1^ step gradient, top to bottom, pre-formed in polyallomer ultracentrifuge tubes for Beckman rotor SW41. Phages in TM (3 mL) were applied to the top of the gradient, and tubes were centrifuged at 100,000 × g for 120 min at 4 °C in a Beckmann Optima XE-90 ultracentrifuge using a SW41 rotor. The phage bands, which usually sediment in the 1.5 g cc^−1^ step, were extracted from the tubes with a syringe, transferred into polyallomer tubes for the SW60 Beckman rotor, and centrifuged for ca. 16 h at 150,000 × g using a SW60 rotor. The phage bands were collected as above, dialyzed 2 x for 20 min against water and 16 h against TM buffer, filtered through 0.22 μm filters and stored at 4 °C.

### Mass spectrometry and SDS-PAGE analysis of N4 virion proteins

Virion proteins were extracted from 7–8 × 10^11^ pfu prepared for cryo-EM analysis as described by mixing 1.5 mL of the phage suspension in TM with 1.5 mL of methanol and 1.125 mL of chloroform. After vigorous mixing, the sample was centrifuged for 5 min at 16,873 × g in a microfuge, and the upper fraction was discarded. The lower fraction and the interface were mixed with 1.3 mL of methanol and centrifuged as above. The protein pellet was dried and resuspended in 6 M urea dissolved in 10 mM Tris-HCl pH 7.4. The viral proteins were analyzed both by 15% SDS-PAGE and Coomassie staining and by mass spectrometry at UNITECH OMICs (University of Milano, Italy) using Dionex Ultimate 3000 nano-LC system (Sunnyvale CA, USA) connected to Orbitrap Exploris^™^ 240^™^ Mass Spectrometer (Thermo Scientific, Bremen, Germany) equipped with nano electrospray ion source. Peptide mixtures were pre-concentrated onto a PepMap 100 – 0.3 × 5 mm C18 (Thermo Scientific) and separated on EASY-Spray column ES902, 25 cm × 75 μm ID packed with Thermo Scientific Acclaim PepMap RSLC C18, 3 μm, 100 Å using mobile phase A (0.1 % (v/v) formic acid in water) and mobile phase B (0.1 % (v/v) formic acid in acetonitrile 20/80, (v/v)) at a flow rate of 0.300 μL min^−1^. The temperature was set to 35°C, and 5 μL samples were injected in triplicate. MS spectra were collected over an m/z range of 375–1500 Da at 120,000x resolution, operating in data-dependent mode, cycle time 3 sec between master scans. HCD was performed with a collision energy set at 35 eV. Polarity: positive. Data were processed using Proteome Discoverer 2.5 software (Thermo Scientific, USA) with the search database set as Escherichia phage N4 (sp_tr_incl_isoforms TaxID=2886925_and_subtaxonomies) (v2024–03-27) and trypsin as the digestion enzyme. The following filters were applied: Protein level, ≥ 2 peptides; Peptide level, Xcorr ≥ 2.2, Rank = 1, Confidence = high; PSMs level: Xcorr ≥ 2.2.

### Molecular cloning, expression, and purification of recombinant proteins

The genes encoding gp50 full length (gp50-FL), gp50-N (1–906), RNAP (residues 998–2103), gp51 (1–644), and gp52 (1–150) were amplified by PCR from N4 DNA and cloned between XhoI and BamHI restriction sites in pET-16b. The constructs were expressed in BL21-AI (Invitrogen), NiCo21(DE3) *E. coli* expression strain (New England Biolabs) or *E. coli* (DE3) omp8 strain supplemented with the proper antibiotics.

The cultures were grown in L.B. medium at 37 °C until an optical density at 600 nm (OD_600_) = ~0.3 when the temperature was dropped to 28 °C until an OD_600_ = ~0.6 and were induced with 0.5 mM IPTG and 0.2% L-arabinose for 3–4 h. In the case of gp52, the culture was induced at OD_600_ = ~1 with 0.5 mM IPTG and 0.2% L-arabinose for 2 h at 37 °C. For gp50-FL, the cell pellet was lysed by sonication in lysis buffer (20 mM Tris-HCl pH 8.0, 50 mM NaCl, 2 mM MgCl_2_, 5% glycerol, 1 mM EDTA, 0.1% Triton X-100, 1 mM PMSF, 20 μg mL^−1^ DNase). After centrifugation at 18,000 rpm for 30 min, 4 °C, the soluble fraction containing the protein was subjected to HiTrap Heparin HP column and washed with wash buffer (10 mM Tris-HCl pH 8.0, 50 mM NaCl, 5% glycerol, 1 mM EDTA) and eluted with buffer B (10 mM Tris-HCl pH 8.0, 1 M NaCl, 5% glycerol, 1 mM EDTA). Fractions containing protein were pooled and buffer-exchanged to wash buffer using an Econo-Pac 10DG column (BioRad). Gp50 tends to reversibly salt out in a buffer containing less than 50 mM NaCl. Next, the protein was subjected to HiTrap DEAE FF (Cytiva) and eluted with buffer B listed above. Finally, the protein was polished by SEC using a Superdex 200 16/60 column (Cytiva) equilibrated with gel filtration buffer (20 mM Tris-HCl pH 8.0, 75 mM NaCl, 2.5% glycerol, 0.5 mM EDTA, 0.0075% DDM, 0.1 mM PMSF). For other gp50 constructs, the cell pellets were lysed by sonication in lysis buffer (20 mM Tris-HCl pH 8.0, 250 mM NaCl, 2 mM MgCl_2_, 5% glycerol, 0.1% Triton X-100, 1 mM PMSF, 20 μg mL^−1^ DNase). After centrifugation at 18,000 rpm for 30 min at 4 °C, the soluble fraction was incubated with Low Density Nickel Agarose beads (GoldBio) for 2 h with rotation at 4 °C. The beads were washed with a wash buffer (20 mM Tris-HCl pH 8.0, 250 mM NaCl, 2 mM MgCl_2_, 2.5% glycerol, 1 mM PMSF, 5 mM imidazole) and eluted with wash buffer containing 20–320 mM imidazole. The fractions containing protein complex were further purified by SEC using a Superdex 200 16/60 column (Cytivia) equilibrated with gel filtration buffer containing 20 mM Tris-HCl, pH 8.0, 100 mM NaCl, 2.5% glycerol, and 0.1 mM PMSF.

For gp50-N (1–906), gp50-RNAP (residues 998–2103), gp51 (1–644), and gp52 (1–150), the protein was isolated from inclusion bodies. The cell pellet was lysed by sonication in lysis buffer (20 mM Tris-HCl pH 8.0, 300 mM NaCl, 4 mM MgCl_2_, 2.5% glycerol, 2 mM EDTA, 0.1% Triton X-100, 1 mM PMSF, 20 μg mL^−1^ DNase). After centrifugation at 18,000 rpm for 30 min, 4 °C, the pellet fraction containing the protein was resuspended in solubilization buffer (20 mM Tris-HCl pH 8.0, 200 mM NaCl, 1% glycerol, 0.35% N-Lauroylsarcosine) and allowed to solubilize by rotating for 1:30 h at room temperature. The supernatant, after centrifugation at 18,000 rpm for 30 min at 4 °C, was incubated with Ni-NTA resin (GoldBio) for 2 h. The resin was packed in a column and washed with 50–100 CV refolding buffer (20 mM Tris-HCl pH 8.0, 200 mM NaCl, 2 mM MgCl_2_, 1% glycerol, 0.05% DDM, 1 mM PMSF) supplemented with 5 mM imidazole. The proteins were eluted stepwise with 20–600 mM imidazole. The eluted protein was placed in Snakeskin^™^ Dialysis Tubing 10k MWCO (Thermo Scientific) and gently agitated in dialysis buffer containing 20 mM Tris-HCl pH 8.0, 100 mM NaCl, 2 mM MgCl_2_, 1% glycerol, 0.05% DDM, 1 mM PMSF overnight at 4°C. After dialysis, the proteins were gel filtrated on a Superose 6 10/300 column (Cytiva) containing dialysis buffer.

For lipid bilayer experiments, gp51 and gp52 were expressed in *E. coli* (DE3) omp8 strain. The final gel filtration buffer was 20 mM HEPES pH 7.4, 100 mM NaCl, 0.05% DDM.

### *In vitro* transcription assay

Each reaction contained a final concentration of 200 μg mL^−1^ of N4 vRNAP protein (or variant) in 40 mM Tris-HCl pH 7.9, 50 mM KCl, 6 mM MgCl_2_, and 0.1 mg mL^−1^ BSA, and 1 mM P1 promoter (underlined) having 30 bp of native DNA sequence on either end (5’-TAGTTGATTGATAAGATACGGACAACATGTCCATAAGTTGCGAAGCAACGTGTAACGTGTACAAGGTGGGGTAGGCTAAGG-3’). These components were mixed and incubated for 5 min at 37 °C. Reactions were initiated by addition of NTPs (1 mM UTP, 500 mM ATP, 500 mM GTP, 10 mM CTP and 10 mCi α−32P CTP). Transcription reactions proceeded for indicated times and were halted by addition of equal volume of formamide loading dye ^47^.

### Reconstitution in membrane nanodiscs

Membrane scaffolding protein 1E3D1 (MSP1E3D1) was expressed in NiCo21(DE3) *E. coli* expression strain (New England Biolabs) supplemented with 35 μg mL^−1^ kanamycin. The cultures were grown in 2xYT medium at 37 °C until OD_600_ = ~0.5, when the temperature was dropped to 28 °C until an OD600 = ~1, and were induced with 0.5 mM IPTG for 3–4 h. The cell pellet was lysed by sonication in lysis buffer containing 20 mM HEPES pH 7.5, 200 mM NaCl, 20 mM MgSO_4_, 1 mM PMSF, and 0.5 mM tris(2-carboxyethyl)phosphine (TCEP). The MSP1E3D1 lysate was clarified by ultracentrifugation at 18,000 rpm for 30 min at 4 °C and the recovered supernatant was supplemented with 50 mM sodium cholate and incubated with Ni-NTA resin (GoldBio) for 2 h with rotation at 4 °C. The resin, after packing in a column, was washed with wash buffer containing 20 mM HEPES pH 7.5, 200 mM NaCl, 20 mM Imidazole, and MSP1E3D1 was eluted with a buffer containing 20 mM HEPES pH 7.5, 200 mM NaCl, 400 mM Imidazole. The eluted protein was placed in Snakeskin Dialysis Tubing 10k MWCO (Thermo Scientific) containing TEV protease (GenScript) and then placed in dialysis buffer consisting of 20 mM HEPES pH 7.5 and 150 mM NaCl. After dialysis, the mixture was repassed through Ni-NTA resin to recapture cleaved His-tag and TEV protease, and the flow-through was collected. Untagged MSP1E3D1 was concentrated to 10 mg mL^−1^ and stored in −80 °C freezer.

Nanodiscs were assembled by mixing purified gp50-N at 5.7 mg mL^−1^ with 16:0–18:1 PC 1-palmitoyl-2-oleoyl-sn-glycero-3-phosphocholine) (POPC) (Avanti Polar Lipids, Inc) at 15 mg mL^−1^ and untagged scaffolding protein MSP1E3D1 at 10 mg mL^−1^, resulting in a molar ratio of 1:500:5. The mixture was incubated for 1 h in the dark at 4 °C before adding 100 mg Bio-Beads SM-2 adsorbents (BioRad) and proceeded for overnight incubation in the dark. The mixture without the Bio-Beads was incubated with Low-Density Nickel Agarose beads (GoldBio) for 30 minutes with rotation at 4 °C. The beads were washed with wash buffer (20 mM HEPES pH 7.5, 100 mM NaCl, 10 mM imidazole) and eluted with wash buffer containing 300 mM imidazole. The mixture was injected onto a Superdex 200 10/300 (Cytiva) column, which was equilibrated with a buffer containing 20 mM HEPES, pH 7.5, and 75 mM NaCl. Throughout the purification process, protein samples were set aside to run on 12.7% SDS-PAGE and 1.5% native agarose gels.

### Vitrification, cryo-EM screening, and data collection

Recombinantly expressed full-length N4 gp50 was vitrified on 300-mesh copper R 1.2/1.3 Quantifoil holey carbon grids. Both the apo and the P1 promoter-bound vRNAP were frozen together. Grids were glow-discharged using a Tergeo-EM Plasma Cleaner for 30 s with power at 20 W. 3 μL of purified protein at 0.5 mg mL^−1^ was applied to grid. Grids were blotted using a Vitrobot Mark IV (FEI), for 5.5 sec using a blot force of 4 before being vitrified immediately in liquid ethane. Purified N4 Virion was vitrified on 300-mesh copper R 2/1 C-Flat holey carbon grids. Grids were glow discharged with identical settings as N4-vRNAP. A total of 6 μL of N4 mature virions at 1 × 10^12^ pfu was applied. A double blotting strategy was used first pipetting 3 μL onto the grid and manually blotting while simultaneously adding the next 3 μL. Following this application, grids were blotted using a Vitrobot Mark IV (FEI), for 5 sec using a blot force of 4 before being vitrified immediately in liquid ethane. Both purified gp50 and N4 grids were screened in house at the University of Alabama at Birmingham Cryo-EM Core, on a 200 kV Glacios 2 equipped with a Falcon 4i detector. Grids for the Apo and P1 gp50 were shipped to SLAC for full data collection on a 300 kV Titan Krios with a Falcon 4i detector. Micrographs were collected with a pixel size of 1.217 Å at 120,000x magnification, using a total dose of 50 e-/Å^2^ and a defocus range of −0.5 μm to −2.5 μm. A total of 13,007 and 5,323 movies were collected for the Apo and P1 gp50, respectively. For the N4 virion, three complete data collections were carried out and merged at the UAB Cryo-EM Core. Identical preparation and settings were used for vitrification and data collection of the virion grids, except for the final collection, where the sample was incubated at 42 °C for 10 minutes before vitrification, in an effort to enrich for empty particles. EPU software was used for data collection using the fast-positioning mode. Micrographs were collected with a pixel size of 1.19 Å at 120,000x magnification, using a total dose of 40 e-/Å^2^ and a defocus range of −0.5 μm to −2.5 μm. Further collection parameters are in Table 1 and Table 2.

### N4 virion bubblegrams

Virions were vitrified as described in the previous section. Grids were imaged at the UAB Cryo-EM core on a 200 kV Glacios 2 equipped with a Falcon 4i detector. Nine movies were collected with a total dose of 250 e-/Å^2^, capturing 39 FVs and 1 EP. Cryo-SPARC was used to fractionate the movies during Patch Motion Correction into 9 static overlapping frames each with a total dose of 50 e-/Å^2^ with 25 e-/Å^2^ size steps between each frame (1^st^ frame: 50 e-/Å^2^ total dose, 50 e-/Å^2^ accumulated dose, 2^nd^ frame: 50 e-/Å^2^ total dose, 75 e-/Å^2^ accumulated dose, 3^rd^ frame: 50 e-/Å^2^ total dose, 100 e-/Å^2^ accumulated dose, etc.). Out of 39 virions, 37 had identifiable tails, and a point was chosen at the interface between the tail and capsid using ImageJ. Next, the center of all visible bubbles was chosen, and the distance between the tail:capsid interface and bubble center was measured on a per virion basis. The Mclust package was used in R to fit the data to a trimodal distribution, and ggplot2 was used to overlay the distribution and the bubble histogram.

### Cryo-EM single particle analysis

All steps of SPA were carried out using cryoSPARC software ^48^ using Patch Motion Correction and Patch CTF Estimation. For the vRNAP reconstruction, blob picking followed by 2D classing and template picking resulted in 2D classes representing four putative species: the RNAP domain by itself, the CTD by itself, and two conformations of the RNAP and CTD in complex. The isolated RNAP domain was resolved with the standard ab initio to non-uniform pipeline. Initial attempts to resolve the three remaining states were difficult due to preferred orientation. A two-class *ab initio* using particles of all states resulted in an isotropic RNAP map and an anisotropic CTD map. A map was reconstructed using Homogenous Reconstruction only, using the RNAP-CTD tight complex particles determined by 2D classification, but with alignments from the isotropic RNAP map generated from the 2-class ab initio. The resulting map from homogeneous reconstruction only had isotropic density for the RNAP domain and anisotropic density for the CTD. Heterogeneous Refinement was performed with six maps. An isotropic RNAP map, an isotropic RNAP-anisotropic CTD map, an anisotropic CTD map, and three “junk” classes generated by running a multi-class ab initio for a single iteration. This Heterogeneous Refinement resulted in maps of similar quality. The mixed iso-RNAP/aniso-CTD map was further subjected to a 25-class 3D classification, where a single class representing 8.7% of the input particles (~12,000 out of ~141,000) had an isotropically resolved CTD that further resolved to 3.8 Å. The RNAP-CTD loose complex was represented by a single class, though it could be refined to high resolution with particles that belonged to the RNAP domain only classes. With a medium-resolution map of the RNAP-CTD complex, templates were simulated using the Create Templates job and then used for template picking, followed by 2D classing to remove obvious junk (carbon edge, ice, etc). The resulting stack of 1.2 million particles was then used for another Heterogeneous Refinement with nine input maps: 3 RNAP domain focused states (High-Res, Med-Res, RNAP-CTD loose complex, 2 RNAP-CTD tight complex states (Isotropic, Anisotropic), 1 CTD isolated map, and three junk classes, which resulted in final particle stacks for the RNAP domain map and the RNAP-CTD tight complex map. During processing of the P1-bound (5’-TGCCTCCCAGGCAGTCAAAAGTTGCGAAGCAAC-3’) gp50, the similarity to the apoenzyme was apparent, and 2D classes and initial maps were nearly identical with the addition of the hairpin DNA promoter. As this was the case, P1 states were solved using a Heterogeneous Refinement job with the input models from the apoenzyme processing and the resulting maps had clear DNA density that was absent from the input models. With the final particle stacks in hand, Reference-Based Motion Correction, Local CTF Refinement, and 3D Classification were used to improve the resolution further, resulting in four final high-resolution maps. For the RNAP-CTD loose complex, we leveraged the high-resolution CTD from the tight complex. Any attempts to resolve this conformation resulted in an isotropic RNAP and an anisotropic CTD. The map of the loose complex from the 9-class Heterogeneous Refinement was aligned and resampled to the tight complex map in ChimeraX ^49^ with respect to the tight complex CTD. A mask was then generated around the CTD in ChimeraX ^49^. The realigned loose complex map was then imported into CryoSPARC ^48^, and the original map and particles were aligned to the imported map using the Align 3D Maps job. The mask around the CTD was used to locally refine the CTD of both the tight conformation and the loose conformation particles, with hopes that the anisotropy of the loose complex CTD could be resolved by the isotropy of the tight complex. The resulting loose complex particles were reconstructed without the tight complex particles, although this time the CTD was isotropic; however, the RNAP was poorly resolved. 3D classing on this particle stack with the CTD-focused alignments resulted in a single class of ~18,000 particles that had the RNAP and CTD equally resolved. The appearance of density missing from the high-resolution CTD from the tight complex (res. 2643–2663), which appears to be critical for the stabilization of the loose complex, gives us high confidence in the reality of the density.

For the N4, virion particles were selected using Blob Picker, followed by the removal of junk particles through 2D classification. High-quality picks were used to train a Topaz model for both full particles and empty particles. The results of Topaz picking yielded 40,673 and 25,795 total full and empty phage particles, respectively. An initial 3D map was generated through *ab initio* reconstruction with icosahedral(I) symmetry enforced. With refined icosahedral capsids, the Symmetry Expansion job was used to expand I symmetry. A cylindrical mask was created in ChimeraX ^49^ and resampled to the capsid map to loosely cover the pentamer at an icosahedral vertex. Using this as a focus mask, 3D Classification was used to sort out the symmetry-breaking vertex with the phage tail. cryoSPARC ^48^ employs only one icosahedral symmetry, I in cryoSPARC ^48^ and I2 by RELION convention (the symmetry axis placed on the two-fold axis at the icosahedral edge-midpoint). Volume Alignment Tool was used to move the five-fold axis into the z-axis, whereby another round of 3D classification was used with C12 symmetry enforced to separate out the symmetry-mismatched phage tails based on portal symmetry. Duplicates were removed, yielding a total particle number of 30,374 and 19,338 for full and empty particles. Using Volume Alignment Tools, both empty and full particles were recentered and re-extracted to the portal to be used for the C12 portal reconstructions, as well as recentered and re-extracted further down the tail to be used for the C6 tail reconstructions. Resolution of the asymmetric tail-gating complex began after a focused 3D classification of a single tail sheath heterodimer. This classification led to two states (detached and displaced), and in both of these states, the poorly resolved density of the plug appeared slightly improved. As we observed no major differences in these features when comparing full and empty particles, both particle sets were combined during these steps. To ensure that the plug was present in both full and empty particles, the stacks were separated and reconstructed using Homogeneous Reconstruction Only, yielding strong density for the plug and all tail components in both full and empty maps. Local CTF refinement and Ewald Sphere correction were used to improve resolution. Subsequent refinements and particle cleanings through 3D Classification led to final particle stacks. Final particle numbers for all maps referenced are indicated in Tables 1 and 2. The final densities were sharpened using *phenix.auto_sharpen* ^50^. Electron density maps were displayed using ChimeraX ^49^.

### Model building and refinement

All *de novo* atomic models presented in this paper were built using Coot ^51^ or ChimeraX ^49^, including the ISOLDE plugin ^52^. AlphaFold3 ^53^ models guided by residue placements from ModelAngelo ^54^ were used as starting templates, and gaps or poorly predicted regions were filled in *de novo* when present. Five maps and resulting models were generated from the full-length vRNAP datasets (Table 1): (**i**) a 2.8 Å map of the recombinant N4 vRNAP with the RNAP and CTD in the tight complex. Residues 1007–3500 (full length 3,500 aa) were modeled using this map. Density for this conformation was continuous, barring a 23-residue linker connecting the RNAP and CTD (2102–2124), and two loops in the CTD: 21 residues from 2643–2663 and 7 residues from 2522–2528. (**ii**). a 2.6 Å map of the recombinant N4 vRNAP with the RNAP domain only, residues 1007–2101. (**iii**) a 4.3 Å map of the recombinant N4 vRNAP with the RNAP and CTD in the loose complex. (**iv**) a 2.9 Å map of the recombinant N4 vRNAP with the RNAP and CTD in a tight complex with the P1 promoter bound, with identical residues modeled in (i). (**v**) a 2.8 Å map of the recombinant N4 vRNAP with the RNAP domain only (1007–2101).

Reconstruction of the N4 virion yielded five maps and corresponding relative atomic models (Table 2). (**vi**) a 3.2 Å C6 averaged map of the full particle tail. Models build using this map were the tail tube (gp54: Full Length), the adaptor (gp67: Full Length), the sheath large subunit (gp65: 1–1184), the sheath small subunit (gp64: Full Length), the N-terminus of the appendage trimer (gp66: ~1–80), and the portal (gp59: 19–693). Inside the tail tube at the portal tail interface, we observed unexpected density, which, through de novo modeling, we identified as the putative outer membrane pore (gp52: 1–16). (**vii**) a 3.6 Å C6 averaged map of the empty particle tail. Models build using this map were the tail tube (gp54: Full Length), the adaptor (gp67: Full Length), the sheath large subunit (gp65: 1–1184), the sheath small subunit (gp64: Full Length), the N-terminus of the appendage trimer (gp66: ~1–80), and the portal (gp59: 19–693). (**viii**) a 2.9 Å C12 averaged map of the full particle portal. The portal (gp59: 19–693) was the sole model refined against this map. (**ix**) a 3.2 Å C12 averaged map of the empty particle portal. As with the prior map, the portal (gp59: 19–693) was the sole model refined against this map. (**x**) A locally refined C1 map of the N4 tail, including the tail-gating complex. Models built from this map include the plug gp53 (res. 9–724) and the final residues of gp65 (res. 1183–1382). All atomic models were refined using several rounds of rigid-body, real-space, and B-factor refinement using *phenix.real_space_refinement* ^55^ and validated using MolProbity ^56^. Refinement statistics are in Table 2.

### Structure analysis, AlphaFold prediction, and bioinformatics tools

All renderings of protein structures and map surface representations were generated using ChimeraX ^49^. Model analysis and inspection were carried out in PyMol ^57^. Structural comparison and identification were done using the Foldseek server ^58^. Binding interfaces were analyzed using PISA ^59^ and PDBsum ^60^. All RMSDs in the Cα position between superimposed structures were calculated using Coot ^61^. Decomposition of vRNAP cryo-EM structure into protein units was carried out using the web server SWORD2 ^31^. Ejection proteins gp51 and gp52 nonameric models were generated using AlphaFold3 ^53^, whereas other monomeric models used in this study were generated with AlphaFold ^62^ and AlphaFold2 ^63^. Membrane insertion was predicted using MemBrain ^34^.

### Lipid bilayer measurements

Lipid bilayer experiments were performed in a custom-made lipid bilayer apparatus as previously described ^64^. Briefly, a Teflon cuvette with 10 mL volume is separated into two compartments (cis- and trans-) by a wall with apertures of approximately 0.3 mm or 1 mm in diameter. Ag/AgCl electrodes were bathed in a 1 M KCl, 10 mM HEPES (pH 7.4) electrolyte solution. The cuvette was primed on both sides of the aperture with 2% diphytanoylphosphatidylcholine (DPhPC; Avanti Polar Lipids) in chloroform. Then, the cuvette was filled with a 10 mL electrolyte solution. Lipid membranes were painted across the aperture from a solution of 1% DPhPC in n-decane with a Teflon loop. Baseline currents were recorded using only electrolyte and detergent-containing buffer to exclude interference from contaminants and membrane-active detergents. The purified gp51 or gp52 proteins were added to both sides of the cuvette, and the reaction was recorded for 15 minutes. Currents were recorded at −10 mV applied potential using a Keithley 428 current amplifier with a rise time of 30 ms and digitized by a computer equipped with Keithley Metrabyte STA 1800 U interface. The data were recorded with Test Point 4.0 software (Keithley). The data were analyzed in SigmaPlot 11.0 (Systat Software) to generate the graphs shown in this paper. The raw data were analyzed using IGOR Pro 5.03 (WaveMetrics) using a macro provided by Dr. Harald Engelhardt.

## Supplementary Material

Supplementary Files

This is a list of supplementary files associated with this preprint. Click to download.

• N4SUP9292025 nal.pdf

• modelvalidationsmerged.pdf

• nreditorialpolicychecklist.pdf

• nrreportingsummary.pdf

## Figures and Tables

**Table 1.** Map and model refinement statistics for vRNAP

**Data Collection Statistics**					
**Specimen**	N4vRNAP - Apo			N4 vRNAP w/ P1 Promoter	
**Facility/Microscope**	SLAC/Titan Krios				
**Detector**	Falcon 4				
**Imaging Software**	EPU				
**Magnification**	120,000x				
**Voltage (kV)**	300 kV				
**Exposure (e^−^/Å^2^)**					
**Exposure Time (sec)**					
**Defocus range/step (μm)**	−0.5 μm to −2.0 μm				
**Pixel size (Å/px)**	1.217 A/px				
**Total movies**		13,007		5,323	
**(frames/movie)**	50				
**Total dose (e−/Å^2^)**	50 e−/Å^2^				
**Refinement Statistics**					
**Entry**	ΔN-vRNAP Tight Complex (1008–3500)	Isolated RNAP (1008–2101)	ΔN-vRNAP Loose Complex (1008–3500)	P1:ΔN-vRNAP Tight Complex (1008–3500)	P1:Isolated RNAP (1008–2101)
**PDB / EMDB entry**	9PNR / EMD-71769	9PNQ / EMD-71768	9PNT / EMD-71771	9PNW / EMD-71774	9PNV / EMD-71773
**Initial particle number**	238,654	531,502	326,271	206,327	239,323
**Final particle number**	221,813	305,193	18,762	128,569	133,659
**Map Resolution (A) at FSC 0.143**	2.8	2.6	4.3	2.9	2.8
**Map Symmetry**	C1	C1	C1	C1	C1
**Initial Model**	4FF3 AlphaFold3 / *de novo*	3C2P AlphaFold3 / *de novo*	9PNR	4FF3 AlphaFold3 / *de novo*	3C2P
**Chains (Residues/DNA)**	1 (2443)	1 (1095)	1 (2443)	2 (2443/20)	2 (1095/20)
Model-to-Map Correlation Coefficient (CC) *	0.84	0.90	0.53	0.86	0.89
**MolProbity / Clash**	1.17 / 3.60	0.99 / 2.19	1.18 / 3.02	1.54 / 4.83	1.24 / 2.71
**R.M.S. deviations**					
**Bond Length (A) / Angles (°)**	0.002 / 0.412	0.002 / 0.397	0.003 / 0.889	0.002 / 0.436	0.002 / 0.431
**Rotamer outliers (%)**	1.32	0.77	1.32	2.39	1.75
**Ramachandran (%) Fav / Allow / Outlier**	98.07 / 1.93 / 0.00	99.09 / 0.91 / 0.00	98.60 / 1.40 / 0.00	98.19 / 1.81 / 0.00	98.90 / 1.10 / 0.00

**Table 2.** Map and model refinement statistics for N4 virion

**Data Collection Statistics**					
**Specimen**	N4 Virion				
**Facility / Microscope**	UAB Cryo-EM Core/Glacios 2				
**Detector**	Falcon 4i				
**Data Collection Software**	EPU				
**Magnification**	120,000 x				
**Voltage (kV)**	200 kV				
**Exposure (e^−^/Å^2^)**					
**Exposure Time (sec)**					
**Defocus range / step (μm)**	0.75–2.0 (0.25) μm				
**Pixel size (Å/px)**	1.19 Å/px				
**Total movies**	24,989				
**Frames / movie**	40				
**Total dose (e−/Å^2^)**	40 e−/Å^2^				
**Refinement Statistics**					
**Entry**	C6 Tail Full	C6 Tail Empty	C12 Portal Full	C12 Portal Empty	Tail-Gating Complex
**Modeled Proteins**	gp54/gp59/gp64/gp65/gp66/gp67	gp54/gp59/gp64/gp65/gp66/gp67	gp59	gp59	gp54/gp53/gp65
**PDB/EMDB entry**	9YF4 / EMD-72876	9YF5 / EMD-72877	9YF8 / EMD-72880	9YF9 / EMD-72881	9YFT / EMD-72905
**Initial particle number**	30,374	19,338	30,374	19,338	49,712
**Final particle number**	16,320	10,265	16,320	10,265	18,434
**Map Resolution (Å) at FSC_0.143_**	3.28	3.65	2.99	3.24	3.61
**Map Symmetry**	C6	C6	C12	C12	C1
**Initial Model**	AlphaFold3 / *de novo*	AlphaFold3 / *de novo*	AlphaFold3 / *de novo*	AlphaFold3 / *de novo*	AlphaFold3 / *de novo*
**Chains (Residues)**	16 (ASU)	16 (ASU)	12	12	15
Model-to-Map Correlation Coefficient (CC) *	0.85	0.85	0.88	0.88	0.68
**MolProbity / ClashScore**	1.48 / 7.29	1.57 / 7.12	1.49 / 5.71	1.18 / 3.93	1.84 / 6.70
**R.M.S. deviations**					
**Bond Length (Å) /Angles (°)**	0.005 / 0.975	0.002 / 0.541	0.005 / 0.930	0.004 / 0.890	0.002 / 0.440
**Rotamer outliers (%)**	1	1.73	1.74	0.92	1.87
**Ramachandran (%) Fav / Allow / Outlier**	97.62 / 2.38 / 0.00	98.07 / 1.89 / 0.05	98.51 / 1.49 / 0.00	98.59 / 1.41 / 0.00	96.12 / 3.88 / 0.00

## Data Availability

Atomic coordinates for phage N4 ΔN-vRNAP Tight Complex, Isolated RNAP, ΔN-vRNAP Loose Complex, P1 bound ΔN-vRNAP Tight Complex, P1 bound isolated RNAP, C6 averaged N4 Full Virion Tail, C6 averaged N4 Empty Particle Tail, C12 averaged Full Virion Portal, C12 averaged Empty Particle Portal, and asymmetric N4 Tail-Gating Complex have been deposited in the Protein Data Bank with accession codes 9PNR, 9PNQ, 9PNT, 9PNW, 9PNV, 9YF4, 9YF5, 9YF8, 9YF9, and 9YFT. The cryo-EM density maps have been deposited in the Electron Microscopy Data Bank with accession codes EMD-71769, EMD-71768, EMD-71771, EMD-71774, EMD-71773, EMD-72876, EMD-72877, EMD-72880, EMD-72881, and EMD-72905.
